# Intergenerational social mobility and leisure-time physical activity in adulthood: a systematic review

**DOI:** 10.1136/jech-2016-208052

**Published:** 2016-12-15

**Authors:** Ahmed Elhakeem, Rebecca Hardy, David Bann, Rishi Caleyachetty, Theodore D Cosco, Richard PG Hayhoe, Stella G Muthuri, Rebecca Wilson, Rachel Cooper

**Affiliations:** 1 MRC Unit for Lifelong Health and Ageing at UCL, London, UK; 2 Centre for Longitudinal Studies, UCL Institute of Education, London, UK; 3 Department of Population Health and Primary Care, Norwich Medical School, University of East Anglia, Norwich, UK

**Keywords:** PHYSICAL ACTIVITY, SPORT, SOCIO-ECONOMIC, Social and life-course epidemiology, SYSTEMATIC REVIEWS

## Abstract

**Aim:**

To systematically review the association between intergenerational social mobility and leisure-time physical activity (LTPA) in adulthood, in order to assess all published evidence relating to the hypothesis that adults socially mobile between childhood and adulthood will have different levels of LTPA than those in the same socioeconomic group across life.

**Methods:**

A systematic review was carried out following the Preferred Reporting Items for Systematic Reviews and Meta-Analyses (PRISMA) guidelines. Studies were identified by searching databases (MEDLINE, Embase, PsycINFO) and reference lists. Eligible studies examined associations between any indicator of social mobility, based on at least one measure of parental socioeconomic position (SEP) and one measure of own adult SEP, and LTPA in adulthood.

**Results:**

13 studies comprising a total of 44 000 participants from the UK, Finland, Sweden, Australia, USA and Brazil were included. Participants were aged 16–70 years and were from population-based surveys, occupational cohorts and primary care registries. Most studies (n=9) used occupational class measures to identify social mobility; education (n=4) and income (n=1) were also used. There was consistent evidence in nine of the 13 studies that stable high socioeconomic groups tended to report the highest levels of participation in LTPA and stable low socioeconomic groups the lowest. Upward and downwardly mobile groups participated in LTPA at levels between these stable groups.

**Conclusions:**

Cumulative exposure to higher SEP in childhood and adulthood was associated with higher LTPA in adulthood. Thus, a potential outcome of policies and interventions which aim to minimise exposure to socioeconomic adversity may be increased LTPA among adults.

**Trial registration number:**

CRD42016036538.

## Background

Regular physical activity improves physical health and mental well-being and reduces risk of chronic diseases including cardiovascular disease.^1–3^ It is therefore important to understand the factors operating across life which may influence participation. Reviews of cross-sectional studies have reported that adults^4–6^ from lower socioeconomic groups tend to participate less frequently in leisure-time physical activity (LTPA) when compared with their more advantaged peers. Systematic reviews of longitudinal studies^7^ ^8^ have subsequently concluded that, despite heterogeneity in findings, lower socioeconomic position (SEP) in childhood tended to be associated with less LTPA in adulthood, and that associations were partly explained by adjustment for adult SEP.

Intergenerational social mobility, that is, changes in the level of SEP of offspring in adulthood when compared with their parents', may also be related to LTPA during adult life. Several alternative hypotheses have been proposed to explain how changes in SEP across life may relate to LTPA in adulthood. For example, adult LTPA may be predominantly influenced by socialisation effects of childhood SEP (origins hypothesis) or mostly by those of current SEP (destination hypothesis).^9^ Alternatively, under the maximisation hypothesis,^9^ ^10^ those experiencing upward social mobility may adopt LTPA levels found in their destination SEP while the downwardly mobile may retain LTPA rates found in their SEP of origin. Accumulation of additive effects whereby higher childhood and adulthood SEP increase probability of participating in LTPA in a cumulative fashion is also possible,^11^ as is effect modification by adult SEP (synergistic/antagonistic effects).^11^

Studies of other cardiovascular disease risk factors generally find evidence of cumulative additive effects.^12–16^ For example, socially mobile study participants from New Zealand had levels of cardiorespiratory fitness at age 26 in-between the socially stable groups.^12^ Lower SEP also tends to cumulatively increase subsequent risks of overweight and obesity.^13–15^ These findings suggest that adults who have a different level of SEP to their parents might have a different probability of participation in LTPA than others whose SEP remains stable between childhood and adulthood. Therefore, a systematic review was carried out to assess all published evidence relating to the hypothesis that adults socially mobile between childhood and adulthood will have different levels of LTPA when compared with those in the same socioeconomic group across life.

## Methods

This systematic review was carried out in accordance with the Preferred Reporting Items for Systematic Reviews and Meta-Analyses (PRISMA) guidelines^17^ and the protocol was registered with The International Prospective Register of Systematic Reviews (PROSPERO) (registration number: CRD42016036538).

### Eligibility criteria

Prospective and retrospective cohort studies published in English examining the association between changes in SEP from childhood to adulthood (ie, intergenerational social mobility) and LTPA in adulthood were included. Eligible measures of intergenerational social mobility were those derived based on at least one measure of childhood SEP (≤18 years) and one comparable measure of adult SEP,^9^ with SEP representing any resource and/or prestige-based measures of position within a societal structure (eg, occupational class, income).^18^ ^19^ Any LTPA outcome^20^ recorded at or after assessment of adult SEP was eligible for inclusion. Excluded were studies with non-LTPA outcomes (eg, exclusively work-related physical activity), LTPA measured before adult SEP and studies of institutionalised participants (eg, care home residents).

### Search strategy and study selection

**Figure 1:**
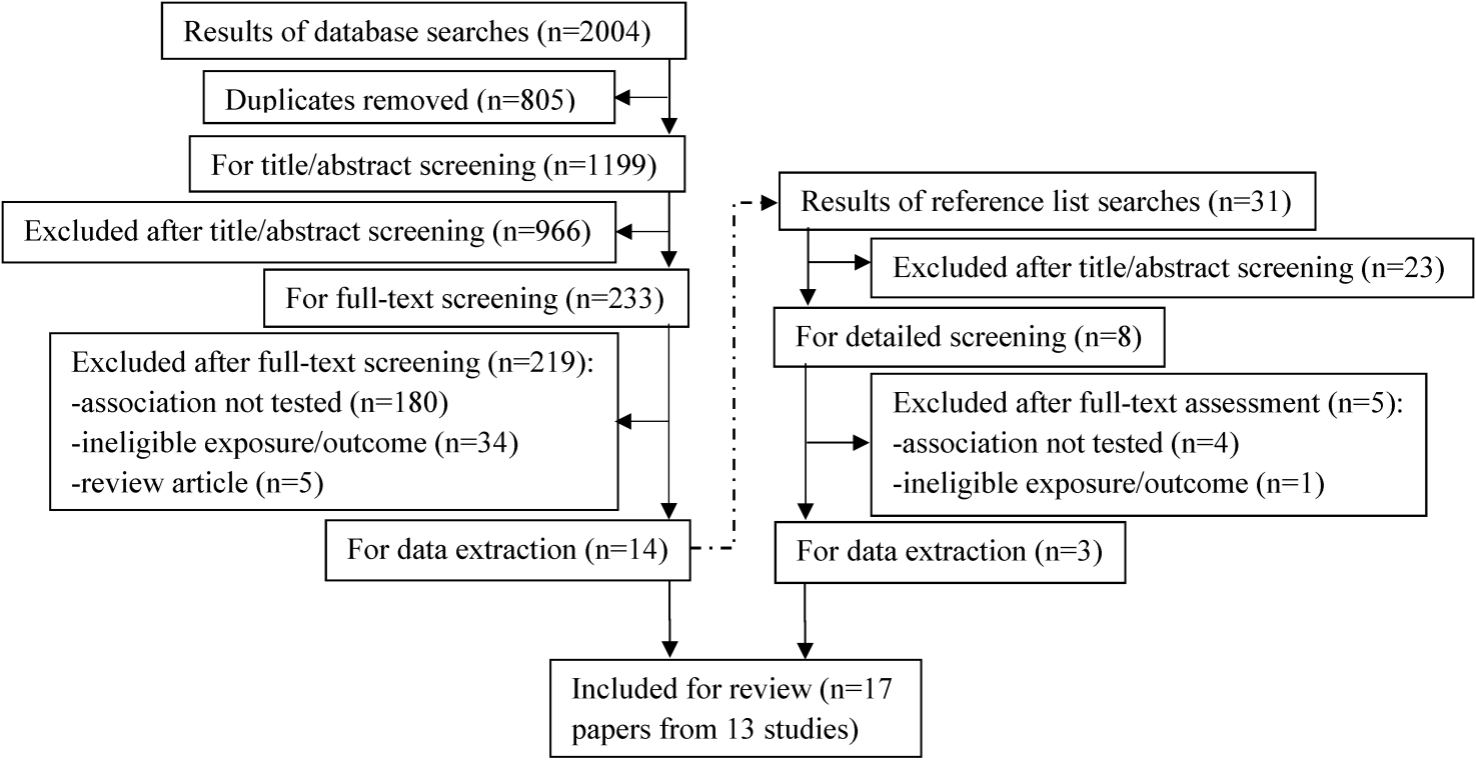
Systematic review flow chart.

Embase (from 1980), MEDLINE (from 1946) and PsycINFO (from 1806) were searched up to October 2015 using keywords (see online supplementary file S1). Duplicates were removed using OvidSP and Endnote. Two independently working reviewers (from AE, RCa, TDC, RPGH, SGM and RW) carried out initial title and abstract screening (to exclude papers that were definitely ineligible) followed by a detailed full-text screening of remaining papers (to exclude papers not meeting all inclusion criteria, with reasons for exclusion recorded). Reference lists of included papers were searched to identify any other eligible studies (figure 1). Any disagreements between reviewers were resolved through discussion and consultation with RCo and RH.

10.1136/jech-2016-208052.supp1 supplementary file S1

### Data extraction and quality assessment

Data extraction and quality assessment of each included paper were carried out by two independently working reviewers using a standardised data extraction form (similar to that developed for our previous review)^7^ ^21^ and a modified Newcastle–Ottawa Quality Assessment Scale^22^ that was developed specifically for this review (see online supplementary file S2). Extracted items were study details (including design, setting and sample size), exposure and outcome details (including how social mobility and LTPA were derived and when these were ascertained), age, sex and birth years of included participants, statistical methods used including adjustment for covariates and lists of potentially eligible papers in reference lists (see online supplementary file S2). All data relating to the association of interest were extracted. Owing to heterogeneity in methods of assessment and analysis between studies, a meta-analysis was not deemed to be appropriate. Quality was judged based on representativeness of the study and source populations, adjustment for covariates, length of follow-up, whether childhood SEP was prospectively or retrospectively ascertained and methods used to assess LTPA. Quality scores were based on the average of two reviewers' ratings (possible values from lowest (0) to highest (9) quality rating).

10.1136/jech-2016-208052.supp2 supplementary file S2

## Results

Of 1199 unique citations retrieved from database searches, 13 studies reported in 17 publications^23–39^ were eligible for inclusion in the review (figure 1).

### Characteristics of included studies

Study characteristics are summarised in table 1. Six were from the UK,^23–31^ two from Finland,^32^ ^33^ one from Sweden,^34^ two from the USA^35^ ^36^ and one study each from Australia^37^ ^38^ and Brazil.^39^ Study participants were mostly from population-based surveys. Others were sampled from primary care registries^24–27^ and three occupational cohorts: 27 workplaces in West of Scotland,^30^ ^31^ and male^35^ and female^36^ physicians from the USA. Age at LTPA assessment ranged from late adolescence to old age with the majority of study participants aged 30 and older (table 1). Birth years were between 1900s and 1980s.

**Table 1:** Characteristics of studies examining associations between intergenerational social mobility and leisure-time physical activity (LTPA) in adulthood: arranged by country

Study name (country) (reference(s))	Description (% female)	Intergenerational social mobility (group definitions)	Physical activity (outcome(s) examined)	Quality scores (average)
MRC National Survey of Health and Development (UK) (Silverwood *et al* 2012).^23^	1946 British birth cohort aged 36–53, n=3847 (49.6%).	Parent's prospectively ascertained and own occupation (always manual (M) or non-manual (NM), upward or downwardly mobile) and education (always lower or advanced, upward or downwardly mobile).	Physical activity latent variables labelled (LTPA: low, gardening and DIY, sports and exercise) and (walking), (cycling) and (sitting).	6, 6 (6)
British Regional Heart Study (UK) (Wannamethee *et al* 1996; Ramsay *et al* 2009).^24^ ^25^	Sample of males aged 52–74 recruited from GP lists in 24 British towns, n=2188 and 5188 (0%).	Parent's recalled and own occupation (always M or NM, upward or downwardly mobile).	Time spent in physical activities such as walking, cycling and sports (active: no description) and (inactive: none or occasionally active).	2, 2 (2); 2, 2 (2)
British Women's Heart and Health Study (UK) (Watt *et al* 2009; Lawlor *et al* 2004).^26^ ^27^	Sample of females aged 60–79 recruited from GP lists in 23 British towns, n=3444 and 3523 (100%).	Parent's recalled and own occupation (always M or NM, upward or downwardly mobile).	Time spent in domestic, recreational and sports activities (low exercise: <2 hours/week) and (low exercise: <1 hours/week).	2, 2 (2); 2, 2 (2)
Scottish Health Survey 2003 (UK) (Popham 2010).^28^	Sample of Scottish residents aged 35–54, n=2770 (% unknown).	Parent's recalled and own occupation: always I/II, IIINM, IIIM or IV/V, upward or downwardly mobile).	Frequency of several types of sports and exercises during previous 4 weeks (participated ≥once in sport/ exercise at moderate/high intensity for ≥15 min/day).	4, 4 (4)
Mid span family Study (UK) (Hart *et al* 2008).^29^	Sample of the 1970s Renfrew/Paisley Study offspring aged 30–59, n=2338 (55.5%).	Parent's prospectively ascertained and own occupation (always M or NM, upward or downwardly mobile).	Frequency of daily physical activity including LTPA (no exercise: not very/at all active in daily activities including at work and active for < once/week or never outside of work).	5, 5 (5)
West of Scotland Collaborative Study (UK) (Blane *et al* 1996; Hart *et al* 1998).^30^ ^31^	Sample of males aged 35–64 employed in 27 Scottish work places, n>5500 (0%).	Parent's recalled and own occupation (always I/II, IIINM, IIIM or IV/V, upward or downwardly mobile) and (always M or NM, upward or downwardly mobile).	Time spent in exercise outside work including walking, gardening and golfing (mean exercise hours/week).	3, 3 (3); 3, 3 (3)
Cardiovascular Risk in Young Finns Study (Finland) (Pulkki *et al* 2003).^32^	9-year follow-up of participants aged 12–21 at baseline, n=1219 (56.4%).	Parent's prospectively ascertained and own education (always low or high, upward or downwardly mobile).	Index of the frequency, intensity and duration of exercise (low exercise score).	6, 6 (6)
Adolescent Health and Lifestyle Surveys (Finland) (Karvonen *et al* 1999).^33^	Sample of young Finns aged 16–18, n=8355 (53.4%).	Parent's prospectively ascertained occupation and index of own education, school attainment and labour market position (always low or high, upward or downwardly mobile).	‘Which of the following describes best your physical activity?’ (Inactive: ‘I do not engage in physical activity at all during my leisure time’).	6, 6 (6)
The Study of Men Born in 1913 (Sweden) (Faresjo *et al* 1994).^34^	Sample of males aged 60 living in Gothenburg in 1963, n=855 (0%).	Parent's recalled and own occupation (always low or high, upward or downwardly mobile).	Exercise levels (no description).	1, 2 (1.5)
The Johns Hopkins Precursors Study (USA) (Kittleson *et al* 2006).^35^	Sample of male physicians aged 40+, n=1131 (0%).	Parental occupation recalled by physicians (always high, upward mobility).	‘How much physical training have you had in the past month? (none, little, moderate and much)’.	1, 1 (1)
Women Physician Health Study (USA) (Frank *et al* 3003).^36^	Sample of female physicians aged 30–70, n=2884 (100%).	Parental education recalled by physicians (always high, upward mobility).	Exercising for at least 30 min 3 times per week.	1, 1 (1)
Childhood Determinants of Adult Health Study (Australia) (Gall *et al* 2010; Cleland *et al* 2009).^37^ ^38^	Follow-up to age 26–36 of the Schools Health and Fitness Survey, n=2047 and 1973 (54.2%; 52.8%).	Parent's recalled and own education (always low or medium or high, upward or downwardly mobile).	Time spent in moderate/vigorous physical activity (persistently inactive or active, increasingly or decreasingly active) and (active: ≥3 hours/week).	4, 4 (4); 3, 3 (3)
Pelotas Birth Cohort 1982 (Brazil) (Azevedo *et al* 2008).^39^	Pelotas birth cohort aged 23, n=4296 (48.5%).	Parent's prospectively ascertained and own income (always poor or non-poor, upward or downward mobility).	Time spent walking, biking, running and in recreational/competitive sports (inactive: <150 min/week).	7, 7 (7)

Parental SEP was prospectively ascertained in five studies (table 1). Changes in occupational class from parent to adult offspring was the most commonly used indicator of social mobility (9/13); educational attainment of parents and their offspring were compared in four studies while income mobility was studied only in a Pelotas birth cohort, Brazil.^39^ Participants were usually classified into four groups depending on whether they were socially mobile upwards/downwards or stable between childhood and adulthood. Four studies present results across more than four mobility groups.^28^ ^30^ ^33^ ^37^ ^38^ Physicians^35^ ^36^ were compared by childhood SEP (ie, implying stable high and upward social mobility). In all studies, LTPA was assessed through self-completed questionnaire or at face-to-face interview and two studies present outcomes combining work-related activity and LTPA.^23^ ^29^ Five studies had low quality rating (range =1–2.5) and four were of medium-to-high quality (range =6–7) (table 1).

### Results of included studies

Most studies present estimates of LTPA as prevalence across stable and mobile socioeconomic groups (table 2). Statistical models were used in some studies and these were either unadjusted or adjusted for age and/or sex. Popham^28^ examined age and sex-adjusted associations with alternating adjustment for childhood and adulthood SEP while findings from Brazil were adjusted for skin colour.^39^ Five studies present separate results for men and women (table 2). Nine of the 13 studies presented some evidence of associations of intergenerational mobility and stability of SEP with LTPA. Results are summarised in table 2 and the following paragraphs.

**Table 2:** Results of studies examining associations between intergenerational social mobility and leisure-time physical activity (LTPA) in adulthood: arranged by country

Study name, country	How results were presented*	Results: prevalence/model estimates*	Adjustment for covariates
MRC National Survey of Health and Development, UK^23^	Prevalence (%) of LTPA (sports and leisure latent class) by occupational and educational mobility.	Occupation: ♂: M/M=24.9, M/NM=35.2, NM/M=27.7, NM/NM=41.5. ♀: M/M=26.5, M/NM=37.9, NM/M=40.0, NM/NM=48.4. Education: ♂: low/low =26.6, low/high =35.1, high/low =34.1, high/high =41.8. ♀: low/low =30.3, low/high =46.2, high/low =37.2, high/high =58.3 (p<0.001 (likelihood ratio test) for both occupation and education and both men and women).	None
British Regional Heart Study, UK^24^ ^25^	Prevalence (%) of LTPA by occupational mobility.	Physically active: M/M=34, M/NM=46, NM/M=35, NM/NM=51 (p<0.05) (Wannamethee *et al* 1996). Physically inactive: M/M=34, M/NM=29, NM/M=32, NM/NM=29 (Ramsay *et al* 2009).	None
British Women's Heart and Health Study, UK^26^ ^27^	ORs of low LTPA by occupational mobility.	ORs of <2 hours/week.: M/NM vs M/M=0.79 (0.66 to 0.94). NM/M vs NM/NM=1.47 (1.05 to 2.06). M/M vs NM/NM=1.55 (1.24 to 1.94) (Watt *et al* 2009). ORs of <1 hour/week: NM/NM=1.00; NM/M=1.67 (1.09 to 2.55), M/NM=1.55 (1.14 to 2.10), M/M=1.90 (1.14 to 2.54) (Lawlor *et al* 2004).	None
2003 Scottish Health Survey, UK^28^	Prevalence (%) and prevalence difference in sports by occupational mobility.	IV and V/IV and V=25.8 (19.0 to 32.6); IV and V/I and II=49.3 (41.1 to 57.6); I and II/IV and V=43.5 (33.2 to 53.8); I and II/I and II=62.8 (58.5 to 67.0). Prevalence difference when compared with those stable in SEP of origin for (1) upwardly mobile: adjusted for parental occupation =9.6 (4.0 to 15.3); adjusted for adult occupation =−6.2 (−11.2 to −1.2) and (2) downwardly mobile: adjusted for parental occupation =−11.0 (−16.5 to −5.5); adjusted for adult occupation =6.2 (0.4 to 12.0).	Age, sex (plus parents’/own adult SEP in model)
Mid span family Study, UK^29^	Prevalence (%) of low physical activity (at work, LTPA and daily activity) by educational mobility.	♂: M/M=16.9 (12.9 to 21.0), M/NM=27.6 (23.0 to 32.2), NM/M=12.9 (5.6 to 20.3), NM/NM=30.8 (25.1 to 36.5). ♀: M/M=20.7 (15.5 to 25.9), M/NM=32.1 (28.4 to 35.8), NM/M=16.3 (6.2 to 26.4), NM/NM=27.0 (22.4 to 31.6).	Age
West of Scotland Collaborative Study, UK^30^ ^31^	Mean exercise hours/week by occupational mobility.	M/M=5.7; 5.9, M/NM=6.3; 6.1, NM/M=6.2; 6.7, NM/NM=6.5; 6.4 (two estimates per group from two measures of adult occupational class: at study screening and at labour market entry) (Hart *et al* 1998). IV and V/IV and V=5.5, IV and V/I and II=5.5, I and II/IV and V=5.2 I and II/I and II=6.5 (Blane *et al* 1996).	Age
Cardiovascular Risk in Young Finns Study, Finland^32^	Mean (SE) low exercise score by educational mobility.	♂: low/low =112.64 (2.72), low/high =115.87 (0.96), high/low =122.59 (5.90), high/high =113.56 (1.53). ♀: low/low =118.45 (1.87), low/high =116.76 (0.8), high/low =120.98 (4.23), high/high =114.89 (1.3) (p=0.3 for ♂ and ♀).	Age
Adolescent Health and Lifestyle Surveys, Finland^33^	Relative risk of no LTPA for socially mobile compared with those stable in SEP of origin.	Downwardly mobile from upper white-collar workers =3.6 (2.0 to 6.4), downwardly mobile from lower white-collar workers =3.7 (2.5 to 5.4), upwardly mobile from blue-collar workers and farmers =0.3 (0.2 to 0.4), upwardly mobile from lower white-collar workers =0.8 (0.5 to 1.3).	Age, sex
The Study of Men Born in 1913, Sweden^34^	Prevalence of low exercise in three occupational mobility groups (text only).	‘The percentage of men who had low exercise levels at the age of 60 was significantly higher among those who had socially moved downwards’ (p (correlation) =0.002) (results for other trajectories not reported).	None
The Johns Hopkins Precursors Study, USA^35^	Prevalence (%) of physical training in male physicians by father's occupational class.	High/high (father with high SEP): none =49.6, little =31.8, moderate/much =18.6. Low/high (father with low SEP): none =50.6, little =31.2, moderate/much =18.2.	None
Women Physician Health Study, USA^36^	Prevalence (%) of regular exercise in female physicians by education of parents.	Mother: < high school =49, high school =50, some college =48, college graduate =48, graduate school =52, medical school =45. Father: < high school =48, high school =48, some college =52, college graduate =49, graduate school =50, medical school =50. Both parents: ≤ high school =48, mix =49, ≥ high school =53.	None
Childhood Determinants of Adult Health Study, Australia^37^ ^38^	Prevalence (%) and change in LTPA by educational mobility.	LTPA at 26–36 years (%): ♂: low/low =31, low/high =36, high/low =47, high/high =50. ♀: low/low =18, low/high =32, high/low =24, high/high =33 (p<0.01 for ♂ and ♀) (Gall *et al* 2010). Relative risk of increasing LTPA from 9–15 to 26–36 years (versus always inactive): ♂: low/low =1.00, low/high =1.49 (1.06 to 2.09), high/low =1.13 (0.76 to 1.69), high/high =1.58 (1.08 to 2.29). ♀: low/low =1.00, low/high =1.38 (1.04 to 1.83), high/low =1.10 (0.79 to 1.54), high/high =1.17 (0.84 to 1.62) (Cleland *et al* 2009).	None (%) Age (model)
Pelotas Birth Cohort 1982, Brazil^39^	Prevalence ratio of low LTPA by income mobility.	♂: non-poor/non-poor =1.00, non-poor/poor =1.32 (1.19 to 1.47), poor/non-poor =1.07 (0.94 to 1.22), poor/poor =1.19 (1.05 to 1.35). ♀: non-poor/non-poor =1.00, non-poor/poor =1.14 (1.08 to 1.20), poor/non-poor =1.06 (0.99 to 1.13), poor/poor =1.18 (1.12 to 1.24), (p<0.001 for ♂ and ♀).	Skin colour

Social mobility is based on change in SEP between parents and adult offspring, that is, intergenerational social mobility.

*For brevity, studies presenting multiple results were not exhaustively extracted to the table. 95% CIs presented unless specified otherwise.

M, manual; NM, non-manual; SEP, socioeconomic position.

In men and women from the 1946 British birth cohort (Medical Research Council (MRC) National Survey of Health and Development),^23^ previously derived latent classes of physical activity types reported between ages 36 and 53^40^ were associated with occupational and educational mobility and stability from parent to offspring (table 2). The highest prevalence of sports and other LTPA was found among those remaining in non-manual occupations (and high educational groups) whereas the lowest prevalence was found among those remaining in manual occupations (and low educational group).^23^ Conversely, the upwardly mobile and those remaining in non-manual occupations and high education groups reported the least walking (during work and leisure) and greatest amount of sitting during the working day.^23^

Differences in physical activity^24^ and inactivity^25^ in leisure time between men from the British Regional Heart Study were greatest between those remaining in non-manual (highest prevalence of LTPA and lowest prevalence of leisure-time inactivity) and manual occupational groups; however, estimates for upwardly mobile men were similar to men stable in non-manual occupational groups.^24^ ^25^ Downwardly mobile women from the British Women's Heart and Health Study were less likely, and upwardly mobile more likely, to be inactive when compared with women stable in the same parental occupational group.^26^ Women from all other groups were also more likely to be inactive when compared with women remaining in non-manual occupations, with the greatest difference found in odds of inactivity for women remaining in the manual occupational group.^27^

More pronounced age and sex-adjusted differences in prevalence of sports and exercise were found across social mobility groups of the Scottish Health Survey 2003 when compared with those reported above.^28^ Further, in models with alternating adjustment for parental and adult SEP, upwardly mobile groups had a higher prevalence of LTPA than those stable in their SEP of origin but lower than those stable in their destination SEP, with the reverse direction found for the downwardly mobile (table 2).^28^ Findings from offspring of the Renfrew/Paisley Study^29^ suggest that the stable non-manual and upwardly mobile groups had lowest levels of daily physical activity (work-related activity plus LTPA) (table 2). The authors report that these findings were due to manual workers performing more daily activities than non-manual workers and that exercise levels outside work were similar for manual and non-manual classes.^29^

Mean reported exercise hours in the West of Scotland Collaborative Study tended to be highest in the stable high and lowest in the stable low groups.^30^ ^31^ Male^35^ and female^36^ US-based physicians who had experienced upward social mobility reported similar levels of LTPA to physicians with equally advantaged parents in terms of occupation class^35^ and education^36^ though exercise prevalence was somewhat higher among the female physicians with two higher educated parents (table 2).^36^ Elsewhere, no associations were found between intergenerational educational mobility and a score based on estimated frequency, intensity and duration of exercise in the Cardiovascular Risk in Young Finns Study.^32^ In contrast, age and sex-adjusted findings from the Adolescent Health and Lifestyle Survey^33^ showed that upward mobility was associated with lower likelihood of leisure-time inactivity among 16–18-year-old Finns and that downward mobility was associated with higher likelihood (table 2). Upwardly mobile children of farmers and blue-collar workers had a lower risk of no LTPA than those stable in the same group while the downwardly mobile from upper white-collar and lower white-collar families had higher risk.^33^ Likewise, downwardly mobile Swedish men born in 1913 performed less exercise than men stable in high SEP.^34^

Australian men and women aged 26–36 remaining in the highest and lowest educational groups between childhood and adulthood had the highest and lowest prevalence of LTPA, respectively, while socially mobile groups had levels in between these stable groups.^37^ Other findings from this cohort showed upwardly mobile men and women, and men stable in the high educational group, were more likely to increase LTPA between ages 9–15 and 26–36 than those stable in the low educational group.^38^ When compared with those always non-poor based on income from a 23-year-old Pelotas birth cohort,^39^ men and women who became poor adults and those who were always poor were both less likely to participate in LTPA (table 2).

## Discussion

### Main findings

This systematic review included findings from 13 studies (reported in 17 publications). It found that intergenerational stability and mobility of SEP was consistently associated with LTPA in adulthood. Of 11 studies that examined intergenerational stability and upward and downward mobility of SEP, nine found similar patterns of association. These suggested that stable high socioeconomic groups reported the highest levels of LTPA and stable low socioeconomic groups the lowest, and that both socially mobile groups participated in LTPA at levels closer to the stable high SEP group. The other two of these 11 studies found no associations. In addition, there were no differences in prevalence of LTPA in the remaining two studies both of which compared physicians who were upwardly mobile with those who had stable high SEP.

### Explanation of findings

The greatest differences in LTPA were between those groups stable in the same SEP and this supports an accumulation of additive effects hypothesis whereby continued exposure to a certain SEP in childhood and adulthood cumulatively alters probability of LTPA.^11^ ^41^ ^42^ This is consistent with studies showing that those with low SEP in childhood and adulthood tend to have the worst health outcomes.^12–16^ ^43–47^ Upwardly mobile groups generally reported more LTPA than those remaining in low SEP of origin which may reflect an adoption of aspirational lifestyle of their destination socioeconomic group.^9^ ^10^ This finding could also be partly due to upwardly mobile individuals working in more sedentary occupations and thus having more energy to participate in LTPA.^23^ The lower occupational physical activity of adults with higher SEP is also likely to explain the null or opposing findings of studies which included occupational physical activity as part of the outcome.^23^ ^29^ Downwardly mobile groups tended to report levels of LTPA more similar to the stable high SEP group than the stable low SEP group which may suggest maintenance of health behaviours adopted in childhood.^7^ ^9^ ^10^ ^48^ ^49^

This review's findings also suggest that transitioning to more advantaged SEP in adulthood may partially offset the influence of low childhood SEP on less LTPA in adulthood shown in previous reviews.^7^ ^8^ However, the relationship between SEP and health of adults is influenced by processes operating during early life which can impact on their health (and related behaviours) and SEP.^41^ ^46^ ^50–55^ Moreover, childhood SEP may be more important for adult health in certain settings than others, for example, a study found father's education to be more important than own education in explaining differences in self-rated health in Eastern when compared with Western Europe.^10^

### Methodological considerations

In assessing the published evidence it is important to consider methodological factors which may influence interpretation. An important limitation of most analyses identified was that they were unadjusted for potential confounders even though certain factors might influence social mobility and LTPA. Related to this, none of the studies described whether the upwardly socially mobile participated in LTPA more, and the downwardly mobile participated in LTPA less, than expected relative to the SEP group they joined which may have indicated selection effects and so helped differentiate genuine effects of social mobility from those due to confounding.^51^ ^52^ In addition, studies tended not to empirically test whether social mobility or other life course models of association best fitted the data.

Most studies relied on recall of childhood SEP which could lead to misclassification due to recall errors and subsequently underestimate associations.^56^ Most studies also relied on binary classifications of childhood and adulthood SEP which may have removed some meaningful variation in patterns of SEP across life. Alternative measures of SEP were rarely considered, for example, household wealth may be an important indicator of SEP in older adults^57^ and it may also be useful to distinguish between types of education.^58^ Social mobility was limited to two time points in all studies; however, duration of exposure to different social positions across life may also influence findings.^59^ Further, all studies examined relative mobility without full consideration of contextual changes. In addition, associations with LTPA may change with age but this could not be examined as most studies included a single measure of LTPA. Lack of difference in LTPA between physicians from different socioeconomic backgrounds may suggest that adult SEP was more closely related to concurrent LTPA but could also reflect insufficient variation in childhood SEP.

All studies relied on participant reports to assess LTPA and although such methods are well suited to capturing LTPA,^60^ ^61^ they can be subject to recall errors, particularly among older participants.^62^ Differential reporting of LTPA by SEP groups is also possible and could bias findings, for example, obesity tends to be more prevalent in lower SEP groups.^13–15^ and obese individuals have previously been found to be more likely to overestimate their levels of physical activity and energy expenditure.^63^ ^64^ Finally, most studies were in high-income countries and thus findings may not be generalisable to low-income or middle-income settings.

### Strengths and limitations of the review

Our systematic review has several important strengths that include the use of a protocol, following of established guidelines, searching of multiple databases and reference lists, and assessment of search results and included studies by pairs of independently working reviewers which helps prevent errors in screening and data extraction. Despite our efforts to locate all published studies, a wider search may have identified additional studies (eg, of economics journals databases and non-English language studies). In addition, we did not search for unpublished studies and we could not formally test for evidence of publication bias as we did not perform a meta-analysis. However, potential publication bias may have been minimised by inclusion of all studies even where associations of interest were not the primary aim.

### Implications of findings

A better understanding of the mechanisms through which socioeconomic circumstances might influence LTPA is required. Studies with repeat assessments of SEP could test alternate hypotheses relating life course SEP to LTPA.^14^ ^16^ ^42^ ^65^ and studies with repeat assessments of LTPA could examine whether associations vary by age. Studies with repeat measures could also use within-person designs as a means of accounting for baseline confounders^66^ and attention should be paid to factors which contextualise SEP such as family and labour market experiences.^46^ Moreover, alternative study designs which offer natural confounder adjustment such as twin studies^67^ ^68^ could help identify the relative importance of early life and adult socioeconomic circumstances for later LTPA, which may help inform appropriate timing of interventions.

The findings of this review suggest that policies and interventions aimed at minimising exposure to socioeconomic adversity^69–73^ could lead to increases in LTPA. These should focus on reducing socioeconomic adversity rather than changing class structure as the latter would result in some people experiencing downward mobility. To this end, improving early life conditions and socioeconomic circumstances may benefit socioeconomic potential and subsequent LTPA.^7^ ^8^ ^69–78^

## Conclusions

This systematic review of intergenerational social mobility associations with adult LTPA included 13 studies and found that those in stable high socioeconomic groups reported the highest levels of participation in LTPA and those in stable low socioeconomic groups the lowest, and that socially mobile groups participated in LTPA at levels between these stable groups. Thus, policies which aim to minimise exposure to socioeconomic adversity may result in improved LTPA levels.

**What is already known on this subject**

- Recent systematic reviews have reported associations between lower socioeconomic position (SEP) in childhood and less leisure-time physical activity (LTPA) in adulthood.
- The association between intergenerational social mobility and LTPA in adulthood has not previously been systematically reviewed.

**What this study adds**

- This is the first systematic review of published evidence on the association between intergenerational social mobility and adult LTPA.
- Cumulative exposure to higher SEP in childhood and adulthood was associated with higher LTPA among adults from different countries.
- Policies which aim to minimise exposure to socioeconomic adversity at any point in life may have the potential to improve LTPA status in adulthood.
