# Supplementary material for: Intergenerational social mobility and leisure-time physical activity in adulthood: a systematic review
Source: J Epidemiol Community Health. 2016 Dec 15;71(7):673–80. doi: 10.1136/jech-2016-208052 (PMC5485757; doi:10.1136/jech-2016-208052)
Supplement: supplementary file S1 [file jech-2016-208052supp001.pdf]

Supplementary file 1 Search terms. (Intergenerational social mobility and leisure-time physical activity in adulthood: a systematic review).

Search Terms
**1.** (physical* activ*)
**2.** (physical* inactiv*)
**3.** (leisure adj3 time)
**4.** (sport*)
**5.** (exercise)
**6.** (walk*)
**7.** (recreational)
**8.** (social mobility)
**9.** (occupational mobility)
**10.** (educational mobility)
**11.** (income mobility)
**12.** (socioeconomic mobility)
**13.** (intergenerational mobility)
**14.** (socioeconomic adj3 trajector*)
**15.** (life* socieoconomic)
**16.** (socieoconomic adj3 patterns)
**17.** (socieoconomic adj3 change)
**18.** (father* adj3 (occupation* or education*))
**19.** (mother* adj3 (occupation* or education*))
**20.** (parent* adj3 (occupation* or education*))
**21.** (father* adj3 (income or manual))
**22.** (mother* adj3 (income or manual))
**23.** (parent* adj3 (income or manual))
**24.** (father* adj3 (social class or social status))
**25.** (mother* adj3 (social class or social status))
**26.** (parent* adj3 (social class or social status))
**27.** (child* adj3 (social class or social status))
**28.** (early-life adj3 (social class or social status))
**29.** (adolescen* adj3 (social class or social status))
**30.** (father* adj3 (socioeconomic or socio-economic))
**31.** (mother* adj3 (socioeconomic or socio-economic))
**32.** (parent* adj3 (socioeconomic or socio-economic))
**33.** (child* adj3 (socioeconomic or socio-economic))
**34.** (adolescen* adj3 (socioeconomic or socio-economic))
**35.** (early-life adj3 (socioeconomic or socio-economic))
**36.** (child* adj3 (deprivation or poverty))
**37.** (early-life adj3 (deprivation or poverty))
**38.** (adolescen* adj3 (deprivation or poverty))
**39.** (adult*)
**40.** (midlife or mid-life or mid-adulthood)
**41.** (old*)
**42.** (later-life)

43. 1 OR 2 OR 3 OR 4 OR 5 OR 6 7 OR 7
44. 8 OR 9 OR 10 OR 11 OR 12 OR 13 OR 14 OR 15 OR 16 OR 17 OR 18 OR 19 OR 20 OR 21 OR 22 OR 23 OR 24 OR 25 OR 26 OR 27 OR 28 OR 29 OR 30 OR 31 OR 32 OR 33 OR 34 OR 35 OR 36 OR 37 OR 38 OR 39
45. 40 OR 41 OR 42 OR 43
46. 44 AND 45 AND 46
47. limit 46 to human
48. Remove duplicates from 47

'root word*': captures alternative word endings. adj3: proximity operator which identifies words within three words of each other.
