# Supplementary material for: Intergenerational social mobility and leisure-time physical activity in adulthood: a systematic review
Source: J Epidemiol Community Health. 2016 Dec 15;71(7):673–80. doi: 10.1136/jech-2016-208052 (PMC5485757; doi:10.1136/jech-2016-208052)
Supplement: supplementary file S2 [file jech-2016-208052supp002.pdf]

Supplementary file 2 Data extraction and quality assessment forms. **(**Intergenerational social mobility and leisure-time physical activity in adulthood: a systematic review).

Modified data extraction form.

A. Reference details							
A1. Record number, 1st author, publication year and title							
A2. Assessor's initials and date of assessment							
B. Study details							
B1. Name of study/cohort							
B2. Birth year(s)							
B3. Design	Prospective cohort		Retrospective cohort				
B3A. If other design please describe:							
B4. Country and setting							
C. Childhood socioeconomic position							
C1. Parental occupation	Yes		No				
C2. Parental education	Yes		No				
C3. If other measures please list							
C4. How ascertained	Prospectively		Retrospectively				
C5. Age recorded							
C6. Age referred to							
D. Adulthood socioeconomic position							
D1. Occupation	Yes		No				
D2. Education	Yes		No				
D3. If other measures please list							
D4. Age recorded							
D5. If any other details please specify							
E. Intergenerational social mobility							
Please describe how intergenerational social mobility was derived:							
F. Physical activity (PA) outcomes							
F1. Type of leisure-time physical activity (LTPA) measured	Sport/exercise		Total LTPA		LTPA plus other PA		
F1A. If other please describe							
F2. How ascertained	Self-reported		Monitored (accelerometer/heart-rate)				
F3. Age ascertained							
G. Available participant numbers							
G1. Baseline	Yes		No		If yes, number		
G2. Excluded	Yes		No		If yes, number		
G3. Lost to follow-up	Yes		No		If yes, number		
G4. Included in analysis	Yes		No		If yes, number		
G5. All accounted for?	Yes		No				
H. Analysis							
H1. How results analysed	Descriptive/ Trend		Logistic regression		Linear regression		Other
H1A. If other please describe							
H2. Included in analysis	Men and women		Men only		Women only		
H3. Only significant results presented?	Yes		No				
I. Summary of results							
I1. Prevalence/Mean difference	Yes		No				
I2. Odds/Risk ratios	Yes		No				
I3. Regression coefficients	Yes		No				
I4. Confidence intervals (CIs)/ P-value/standard errors (SE)	Yes		No				
I5 If other please describe							
J. References for screening							
J1. Please list potentially eligible references/citations from reference list							
K. Effect estimates							

Association tested	Number analysed	Type of effect estimate, category comparison/value of unit change and results	95% CI; SE; p-value	Covariates included in analysis
1.				
2.				
3.				

Amended Newcastle-Ottawa Quality Assessment Scale.

Note: A study can be awarded a maximum of two stars for each numbered item – (except number 5).

A. Selection

1) Representativeness of the exposed cohort

a) Truly representative of the source population. ✵ ✵
b) Somewhat representative of the source population. ✵
c) Selected group of users e.g. nurses, volunteers.
d) No description of the derivation of the cohort.

2) Ascertainment of childhood socioeconomic position (SEP)

a) Prospectively from parents/participants when aged ≤18 years. ✵✵
b) Retrospectively collected with attempts to reduce recall bias (e.g. life-grid and structured interview techniques). ✵
c) Retrospectively collected without attempts to reduce recall bias.
d) No description.

B. Comparability

3) Comparability of cohorts on the basis of the design/analysis

a) Study controls for age/sex. ✵
b) Study controls for any additional relevant factors (e.g. childhood health). ✵
c) Only unadjusted results presented.

C. Outcome

4) Assessment of physical activity

a) Monitor device (heart-rate/accelerometer). ✵ ✵
b) Self-reported using validated questionnaire/diary/interview. ✵
c) Self-report.
d) No description.

5) Adequacy of cohort follow-up

a) Complete follow up - all subjects accounted for. ✵
b) Subjects lost to follow up unlikely to introduce bias (≥75% follow-up or description provided of those lost). ✵
c) <75% follow-up and no description of those lost.
d) No statement.
